# *Chrysanthemum indicum* L. ameliorates muscle atrophy by improving glucose tolerance in CT26-induced cancer cachexia

**DOI:** 10.3389/fphar.2024.1455805

**Published:** 2024-11-18

**Authors:** Gahee Song, Minji Choi, Woo Yong Park, Sang Hee Kim, Wenjun Jiao, Ja Yeon Park, Kwang Seok Ahn, Hyun Jeong Kwak, Jae-Young Um

**Affiliations:** ^1^ Department of Pharmacology, College of Korean Medicine, Kyung Hee University, Seoul, Republic of Korea; ^2^ Kyung Hee Institute of Convergence Korean Medicine, Kyung Hee University, Seoul, Republic of Korea; ^3^ Department of Science in Korean Medicine, Graduate School, Kyung Hee University, Seoul, Republic of Korea; ^4^ Department of Bio and Fermentation Convergence Technology, Kookmin University, Seoul, Republic of Korea

**Keywords:** cancer cachexia, muscle atrophy, glucose intolerance, glucose transport 4, *Chrysanthemum indicum L*., linarin

## Abstract

**Introduction:**

Cancer cachexia is associated with various metabolic mechanisms such as inflammatory response, insulin resistance, and increased muscle proteolysis. However, effective treatment methods have not yet been standardized. *Chrysanthemum indicum L*. (CI) is a perennial plant belonging to the Asteraceae family, and its flowers have been used for the treatment of headaches, colds, and rhinitis in Asia.

**Methods:**

This study investigated the effect of CI on cancer cachexia. We subcutaneously injected CT26 colon cancer cells (5 × 10^5^ cells/mouse) into the right flank of BALB/c mice. After 1 week, the mice were orally administered vehicle, CI (100 mg/kg), or Celecoxib (50 mg/kg) for 3 weeks.

**Results:**

CI improved loss of body weight and impaired glucose tolerance, but celecoxib did not recover the body weight and glucose intolerance. CI not only improved the decreased myofiber diameters but also inhibited muscle protein degradation factors, MAFbx and MuRF1. CI also increased cellular membrane GLUT4 in CT26 conditioned medium-treated C2C12 myofibers and cancer cachexia-induced mice. Furthermore, we found that linarin, a constituent of CI, was responsible for the improvement of muscle atrophy.

**Conclusion:**

Our findings indicate that CI can ameliorate muscle atrophy by improving glucose uptake, suggesting that CI could be a therapeutic agent for cancer cachexia.

## 1 Introduction

*Chrysanthemum indicum L.* (CI) is a perennial plant belonging to the Asteraceae family and is most widely used as a tea and medicine in Asia, including Korea, China, and India (Youssef et al., 2020). Currently, the clinical applications of CI are extensive, with the plant being used in the treatment of headaches, colds, rhinitis, inflammation, antibacterial, hypertension, and respiratory diseases (Karnwal, 2022; Shahrajabian et al., 2019). To date, over 190 chemical components have been isolated and identified in this plant, including flavonoids, terpenoids, phenylpropanoids, and phenolic acids (Dong et al., 2017; Matsuda et al., 2002). Extracts or monomeric compounds of CI have been reported to have several pharmacological activities, including anti-inflammatory, anti-oxidative, anti-pathogenic microbial, anti-diabetic, and immune regulatory effects (Adisakwattana et al., 2012; Cheon et al., 2009; Lee et al., 2018; Li, 1981). However, the therapeutic potential of CI against cancer cachexia has not been sufficiently evaluated.

Cancer cachexia is a multifactorial syndrome associated with various metabolic mechanisms, including inflammatory response, insulin resistance, and increased muscle proteolysis (Setiawan et al., 2023). The diagnostic criteria for cancer cachexia are rapid weight loss of >5% or >2% and a BMI of less than 20 kg/m^2^ in sarcopenic individuals (Blum et al., 2014). Depending on the type of cancer, the prevalence of cancer cachexia was 87% in pancreatic and gastric cancer patients, 61% in colon, lung, and prostate cancer patients, and 40% in breast, sarcoma, and leukemia patients (Ni and Zhang, 2020). Cancer cachexia accounts for 20% of all cancer-related deaths and is a sign of a negative prognosis (Fearon, 2008), and yet, effective cancer cachexia treatment methods have not yet been standardized. According to ASCO guidelines, pharmacologic approaches of interest for cancer cachexia are appetite stimulants (cannabis and cannabinoids, corticosteroids, cyproheptadine, and megestrol acetate), anabolic agents (anamorelin, androgens, or selective androgen receptor modulators), cytokine inhibitors (hydrazine sulfate, thalidomide, and tumor necrosis factor inhibitors), and others [adenosine triphosphate, insulin, mirtazapine, melatonin, nonsteroidal anti-inflammatory agents (NSAIDS), and olanzapine] (Roeland et al., 2020; Roeland et al., 2023). There are currently no FDA-approved medications to treat cancer cachexia.

Muscular atrophy is one of the key features seen in patients with cancer cachexia, and factors involved in muscle atrophy could be a major therapeutic target for cancer cachexia. During the development of cancer cachexia, skeletal muscle proteins undergo metabolic imbalance due to reduced synthesis and increased degradation (Fearon et al., 2011). These changes eventually lead to muscle atrophy, and an important pathway in the process of skeletal muscle protein degradation is the ubiquitin-proteasome system (UPS). Two E3 protein ligases, muscle atrophy F-box protein (MAFbx) and muscle ring finger protein 1 (MuRF1), have been shown to activate protein degradation in muscle atrophy (Rom and Reznick, 2016). Cancer cachexia patients with skeletal muscle atrophy show consistent activation of MAFbx and MuRF1 (Sukari et al., 2016). They are regulated by a variety of factors and mechanisms such as insulin-like growth factor-1 (IGF-1), phosphatidylinositol-3 kinase (PI3K), protein kinase B (Akt), p38 mitogen-activated protein kinase (MAPK), nuclear factor-kappa B (NF-κB) pathways, proinflammatory cytokine, and insulin (Fearon et al., 2012; Rom and Reznick, 2016).

The presence of insulin resistance has been observed in patients with various types of tumors, and changes in glucose tolerance are associated with cachexia symptoms in cancer patients (Heber et al., 1982). Because skeletal muscles are mainly responsible for insulin-stimulated glucose consumption, insulin resistance in muscles can affect systemic metabolism (DeFronzo and Tripathy, 2009). Patients with cancer cachexia also have elevated plasma glucose, insulin resistance, and excess catabolism. Generally, a high amount of blood glucose leads to increased insulin production. Overproduction of insulin causes the inactivation of PI3K/Akt/mTOR pathway and finally results in insulin resistance. Because insulin maintains the vitality of organs and regulates the metabolism of glucose, protein, and lipids, insulin insensitivity decreases glucose uptake in the organs and results in the loss of skeletal muscles and adipose tissues (Masi and Patel, 2021). Additionally, many molecular studies have demonstrated that insulin-stimulated muscle glucose uptake is highly susceptible to insulin resistance due to impaired GLUT4 translocation (O’Neill et al., 2011; Wojtaszewski et al., 1999). The development of muscle wasting in cancer cachexia patients is related to insulin resistance (Norton et al., 1984). Thus, the present study aimed to explore the effect and the underlying mechanisms of CI on cancer cachexia using CT26 colon cancer cells-inoculated BALB/c mice and conditioned medium (CM)-treated C2C12 cells, focusing on the ability of CI to ameliorate cancer cachexia by controlling insulin resistance in muscles.

## 2 Materials and methods

### 2.1 Reagents

Low glucose DMEM was purchased from WelGENE (Daegu, Korea). DMEM, Penicillin–streptomycin, fetal bovine serum (FBS), and horse serum (HS) were purchased from Gibco (Grand Island, NY, United States). Celecoxib was purchased from LKT Labs (St. Paul, MN, United States). Luteolin, chlorogenic acid, and linarin were purchased from ChemFaces (Hubei, China), and these was diluted using dimethyl sulfoxide (DMSO). Antibody information is provided in Supplementary Table S1.

### 2.2 Preparation of the CI

CI was purchased from Omniherb (Daegu, Korea). CI was extracted in hot water at 100°C for 3 h, followed by filtering using F1003 grade filter paper (CHMLAB Group, Barcelona, Spain). After freeze drying in a vacuum, it was diluted to a concentration of 500 mg/mL using distilled water. The solution was filtered through a 0.22 μm syringe filter and then stored at −20°C for further use.

### 2.3 Animal experiments

**FIGURE 1:**
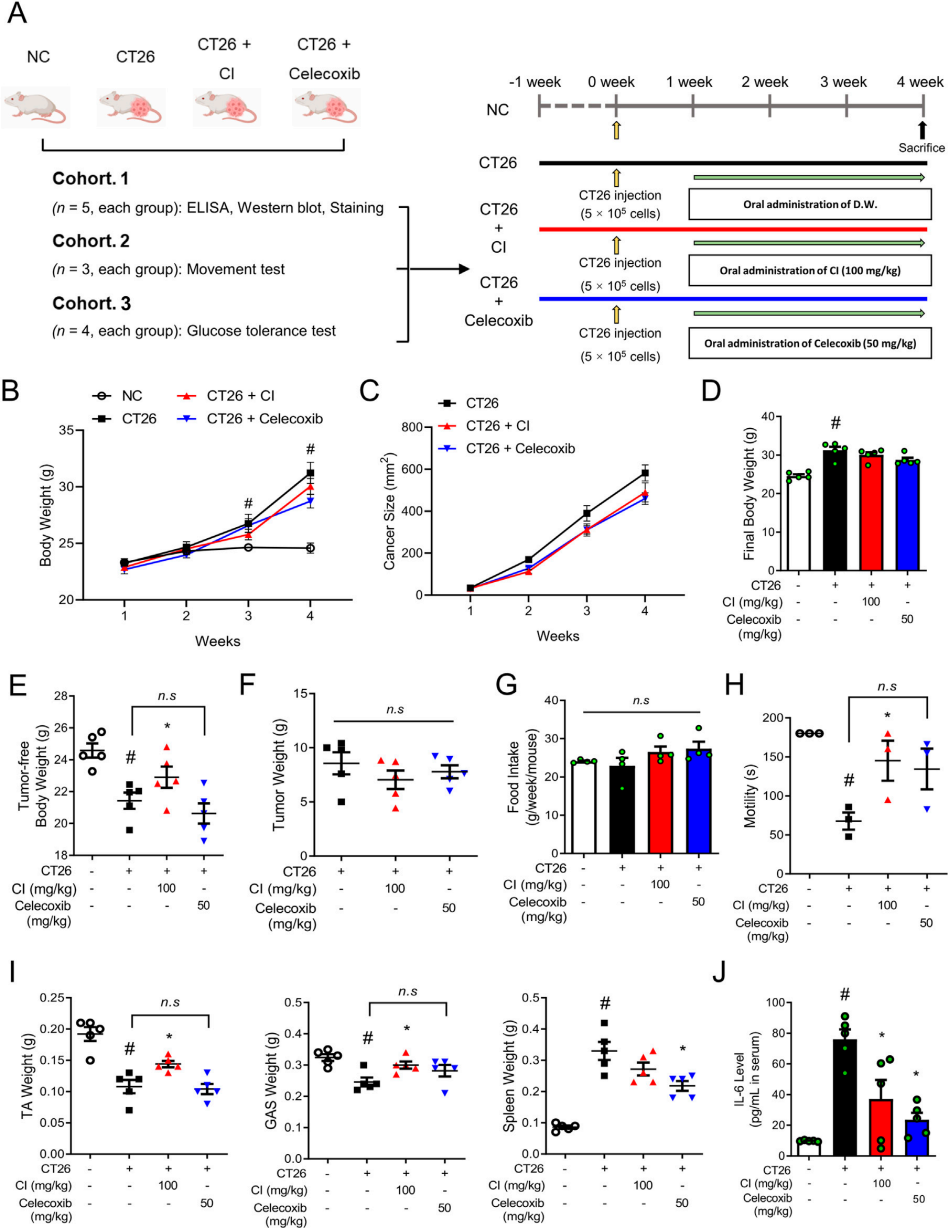
CI increased the tumor-free body weight and skeletal muscle motility in CT26-induced cachectic BALB/c mice. **(A)** Experimental scheme of the *in vivo* study is shown. The BALB/c mice were subcutaneously inoculated with 5 × 10^5^ CT 26 cells in the dorsal region, except for the NC group. Oral administration of CI (100 mg/kg/d) and celecoxib (50 mg/kg/d) were started 1 week after the cancer injection. The control groups (NC and CT26 group) were administered distilled water. **(B, C)** Weekly measurements assessed changes in **(B)** body weight and **(C)** tumor size (*n* = 5/group). **(D)** Body weight was measured immediately before sacrifice (*n* = 5/group). **(E)** Tumor-free body weight was calculated by subtracting the tumor weight from the body weight (*n* = 5/group). **(F)** Tumor weight was measured (*n* = 5/group). **(G)** Food intake was measured every week. **(H)** Motility was measured with a rotarod test (*n* = 3/group). **(I)** Tissue weights of the TA, GAS, and spleen were measured. **(J)** Serum IL-6 level was measured (*n* = 5/group). All data are expressed as the mean ± S.E.M. Statistical differences were calculated by two-way or one-way ANOVA and Tukey’s test. ^#^ *p* < 0.05 vs. NC mice; *p < 0.05 vs. CT26 mice. CI, *Chrysanthemum indicum*; NC, normal control; TA, tibialis anterior; GAS, gastrocnemius.

Three individual animal cohorts were used in the *in vivo* study. The first cohorts (*n* = 5 per group) were used for tissue and serum collection and various biochemical tests, such as ELISA, Western blot, and staining, whereas the second cohort was used for a behavioral test (*n* = 3 per group), and the third cohort was used for the glucose tolerance test (*n* = 4 per group). Detailed information of these animal cohorts and the entire protocol of the experiment is presented in Figure 1A. Male six-week-old BALB/c mice were purchased from Daehan Biolink Co. (Eumsung, Korea) and maintained for 1 week prior to the experiments with a laboratory diet and water *ad libitum*. Cancer cachexia was induced as described in our previous study (Song et al., 2024). All mice were randomly divided into the normal control (NC) group and CT26 group to ensure similar average body weight, which were subcutaneously injected with PBS or 5 × 10^5^ CT 26 colon cancer cells. One week after the tumor injection, the mice in the CT26 group were randomly divided based on average tumor volume and body weight into three groups: a CT26 group, a CT26 group administered CI (100 mg/kg) denoted as the CI group, and a CT26 group administered celecoxib (50 mg/kg) denoted as the celecoxib group. The experiment group was given orally, while the control groups (NC and CT26) were administered distilled water, five times/week for 3 weeks. The dosage of the CI and celecoxib administered to the mice was determined by referring to previous studies and human administration (Gonçalves et al., 2022; Khaleel et al., 2023; Kim et al., 2021; Lee et al., 2019; Sakr et al., 2021). Body weight and food intake were measured once per week. Tumor size was calculated every week based on the formula width x length using an Absolute digimatic caliper (Mitutoyo, Kawasaki, Japan). The relationship between tumor weight and volume can be calculated using the formula 0.52 × tumor length × tumor width^2^ (Chen et al., 2020). Each drug was administered for 3 weeks, and the mice were sacrificed by cervical dislocation under CO_2_ asphyxiation 24 h following the final drug administration. Blood samples were obtained from the caudal vena cava. The blood-containing tubes remained at room temperature for 1 h and then were separated by centrifuging at 3,000 × g for 20 min at 4°C. The serum was stored at −80°C until used in assays. The tissues of tibialis anterior (TA), gastrocnemius (GAS), tumor, and spleen were collected and weighed. Some tissues were used immediately or frozen at −80°C or fixed in 10% formalin. All procedures in the animal experiments were performed after receiving approval from the Animal Care and Use Committee of the Institutional Review Board of Kyung Hee University [KHUASP (SE)-19-405]. All analyses were conducted in an investigator-blinded fashion.

### 2.4 Movement test

At 3 weeks after the drug treatment, mice were subjected to a rotarod test (Choi et al., 2018; Song et al., 2024). A mouse was placed on a rotating rod (rota-rod) with a 4 cm diameter at 15 rpm/s for 3 min. The latency to fall off was recorded by magnetic trip plates. Mice were acclimated to the rota-rod for 5 days before the test. The experiment was carried out in a quiet room by blinded researchers.

### 2.5 Analysis of serum interleukin 6 (IL-6) and insulin levels

Mice were sacrificed 24 h after the final drug administration, and blood samples were collected for the assessment of biochemical parameters. The levels of serum IL-6 and insulin were measured using an enzymatic colorimetric method by commercial ELISA kits (IL-6: R&D Systems, Minneapolis, MN, United States; insulin: RayBiotech, Gwinnett County, GA, United States, respectively) based on the manufacturer’s instructions.

### 2.6 Oral glucose tolerance test (OGTT)

OGTT was performed as previously described (Kang et al., 2020). For the oral glucose tolerance test (OGTT), mice were orally administered glucose (2 g/kg of body weight) in sterilized water after fasting for 16 h at 3 weeks after the dug treatment. Blood glucose was measured from the tail (by removing the distal 2 mm of the tail) at 0, 5, 20, 40, 60 and 120 min after delivering the gavage using a glucometer (Accu-Chek Performa device, Roche Diagnostics, Mannheim, Germany).

### 2.7 Hematoxylin and eosin (H&E) staining

The tissue was washed in PBS and fixed in 10% formalin for 3 days. GAS was cut through the center of the muscle as much as possible. The tissue and cells were washed in PBS and fixed in 10% formalin for 3 days. Then, the tissues were embedded in paraffin. Embedded tissues were cut into 5 μm sections, and the tissue sections were deparaffinized with xylene and rehydrated in ethanol/water. The cells were washed in PBS and fixed in 10% formalin for 1 h. The cells proceeded directly to the next process without this process. The sections were stained with H&E and then examined and measured using an EVOS M7000 Imaging System (Thermo fisher Scientific). The average muscle fiber cross-section area were calculated using the ImageJ software program (National Institute of Health, Bethesda, MD, United States). Measurements were taken from randomly selected areas, with at least three independent images analyzed for each sample.

### 2.8 Protein extraction and western blot analysis

The tissues and cells were lysed with lysis buffer (Cell Signaling Technology) on ice for 30 min and then centrifuged at 13,000 rpm for 30 min at 4°C. The proteins in the lysates were quantified into equal amounts with a protein assay reagent (Bio-Rad Laboratories, CA, United States). Then, the proteins were prepared in a 5× sample buffer, resolved by sodium dodecyl sulfate (SDS)-polyacrylamide gel electrophoresis, and transferred onto a polyvinylidene difluoride (PVDF) membrane. The membranes were blocked in 5% skim milk and incubated with the primary antibody (1:500–1,000) overnight at 4°C, and then incubated with the appropriate horseradish peroxidase (HRP)-conjugated secondary antibody (1:10,000) (Jackson Immuno Research Laboratories, Inc.) for 1 h at room temperature. The proteins were visualized with the ECL advance kit. The information on primary and secondary antibodies used in the experiments is provided in Supplementary Table S1.

### 2.9 Immunofluorescence (IF) staining assay

For IF staining assays, the tissues and cells were fixed with 10% formalin and permeabilized with 0.2% Triton X-100 (Sigma-Aldrich) in PBS for 10 min; thereafter, nonspecific binding sites were blocked using PBS with 1% BSA (Calbiochem, San Diego, CA, United States). The tissues and cells were then incubated with the indicated primary antibodies (1:100 in 5% BSA) overnight at 4°C. After washing, the cells and tissues were incubated with Alexa Fluor 488-conjugated secondary antibody (1:500; Thermo Fisher Scientific) for 1 h at 4°C. Fluorescence signals were imaged with a ZEISS LSM 980 (Carl Zeiss AG, Oberkochen, Germany) at the Core Facility for Supporting Analysis and Imaging of Biomedical Materials at Wonkwang University supported by National Research Facilities and Equipment Center. The fluorescence density was quantified with the ImageJ software (NIH). The information on primary and secondary antibodies used in the experiments is provided in Supplementary Table S1.

### 2.10 Cell culture, differentiation, and preparation of conditioned medium (CM)

To prepare cancer CM, CT26 carcinoma cells were maintained in growth media (10% FBS-containing DMEM), plated in 100-mm culture dishes (1 × 10^6^ cells/dish), and incubated in growth media for 24 h. After washing with PBS, the cells were replaced with serum-free DMEM and incubated for 24 h to exclude the serum inflammatory factors. The CM was collected and filtered using a 0.22 µm syringe filter and diluted in a fresh medium for further use.

Mouse mitotic C2C12 mouse myoblasts (CRL-1772) were purchased from the American Type Culture Collection (Manassas, VA, United States). The C2C12 myoblasts (passage 3–6) were plated on low glucose DMEM containing 10% FBS in 6-well plates. For differentiation into myofibers, 90% of the confluence cells were cultured in a differentiation medium (DM, 2% HS-containing low glucose DMEM) for 3 days. And then to make the cancer-induced muscle atrophy model, differentiated myofibers were treated with culture medium supplemented with 50% CT26 CM and CI (100 and 250 μg/mL) for 24 h. Cell culture, differentiation and preparation of CT26 conditioned medium was performed as previously described (Song et al., 2024).

### 2.11 Cell viability assay

The C2C12 myoblasts were seeded (2 × 10^4^ cells per well) onto 96-well plates and incubated for 24 h followed by incubation with various concentrations of CI (1–1,000 μg/mL), Linarin (1–20 μM), Luteolin (1–50 μM), and chlorogenic acid (1–50 μM) in a culture medium for an additional 24 h. Cell viability was determined using the Quanti-MAX WST-8 assay (Biomax, Seoul, Korea) following the manufacturer’s instructions. Absorbance was measured at 450 nm with a VERSAmax microplate reader (Molecular Devices, CA, United States).

### 2.12 RNA isolation and real-time reverse transcription polymerase chain reaction (qPCR)

RNA isolation and real-time RT-PCR were performed as previously described (Park et al., 2021). Briefly, the total RNA was extracted with a GeneAll RiboEx total RNA extraction kit (GeneAll Biotechnology, Seoul, Korea), and cDNA synthesis was performed with a Maxime RT PreMix Kit (iNtRON Biotechnology, Seoul, Korea). The qPCR was performed with a SYBR Green Power Master Mix (Applied Biosystems, Foster City, CA, United States) and a Step One Real-Time PCR System (Applied Biosystems). The relative gene expressions were calculated based on the comparative CT method with the StepOne software v2.1 (Applied Biosystems). The primer information is provided in Supplementary Table S2.

### 2.13 Glucose uptake assay

Glucose uptake was measured with the 2-NBDG assay, which is a fluorescent-D glucose analogue (Yu et al., 2022). The C2C12 myoblasts were plated on DMEM containing 10% FBS in 96-well black plates and differentiated into myofibers. They were cultured in serum-free low glucose DMEM to cause starvation. After 15 h, myofibers were treated with 50% CM (CT26) and CI (100 and 250 μg/mL) for 1 day. The myofibers were incubated with 20 μM 2-NBDG (Cayman Chemical Company) for 30 min at 37°C. 2-NBDG uptake was stopped by removing the culture medium and washing with PBS. Then, the cells were lysed with lysis buffer (Cell Signaling Technology). The level of 2-NBDG was measured with the GloMax Discover microplate reader (excitation 466 nm, emission 540 nm; Promega Corporation, Madison, WI, United States).

### 2.14 Oxygen consumption rate (OCR) assay

OCR was measured with an Extracellular Oxygen Consumption Assay ab197243; (Abcam, NY, United States) according to the manufacturer’s instructions. Briefly, C2C12 cells on a 96-well black plate were completely differentiated to myofiber and with 50% CM (CT26) and CI (250 μg/mL) for 1 day. After the addition of an extracellular O_2_ consumption reagent (10 μL) to each well, one drop of the provided high sensitivity mineral oil was added to seal the well. The plates were read at 365 nm for excitation and 660–720 nm for emission using the GloMax Discover microplate reader (Promega Corporation, Madison, WI, United States).

### 2.15 Liquid chromatography/mass spectrometry (LC/MS) analysis

We used LC/MS analysis to identify the chemical profiling of CI, which was performed as previously described (Park et al., 2023). Chromatographic separation of the aliquots was done with the Agilent 1,290 infinity LC (Agilent Technologies, Palo Alto, United States) using an Agilent Eclipse Plus C18 column (2.1 × 50 mm, 1.8 µm) and a mobile phase composed of 0.1% formic acid in water (A) and 0.1% formic acid in acetonitrile (B). The gradient program was as follows: 0–3 min, 5% B; 3–13 min, 5%–80% B; 13–15 min, 80% B; 15–17 min, 80%–5% B; and 17–20 min, 5% B. The flow rate was 0.3 mL/min, and the injection volume was 1 μL, which was injected into the column with a thermostatted HiP-ALS autosampler. Separated peaks were analyzed with an Agilent 6550 Q-TOF (Agilent Technologies), which provided high-resolution mass measurements. The instrument was equipped with a Jet Stream ESI source. The ESI spray voltage was set to 4,000 V for the positive ion mode and 3,500 V for the negative ion mode. Mass spectra were acquired in the 100–1,000 m/z range.

### 2.16 Statistical analysis

All data are expressed as mean ± standard error of the mean (SEM) of independent experiments. A statistical analysis was performed using GraphPad Prism 8 (GraphPad Software, San Diego, CA, United States). The differences between the two groups were assessed using an unpaired one-tailed Student’s *t*-test and a subsequent *post hoc* one-tailed Mann-Whitney *U* test. One-way analysis of variance (ANOVA) and Tukey’s test were carried out to assess and compare the differences among more than two groups of independent samples. Values of *p* ^*^ < 0.05 were considered statistically significant.

## 3 Results

### 3.1 CI reduces skeletal muscle weight loss in a cancer cachexia mouse model

To ascertain whether CI has an impact on cancer cachexia, we conducted *in vivo* experiments using CT26 colon cancer cells-inoculated BALB/c mice as a cancer cachexia mouse model (Figure 1A). Two weeks after the CT26 cancer cell injection, the body weight of mice increased in parallel with tumor size, as compared to the NC group (Figures 1B, C). The final body weights of the CI and celecoxib groups were not significantly different from those of the CT26 group (Figure 1D). However, the tumor-free body weight was significantly increased to 22.91 ± 1.49 g only in the CI group compared to 21.43 ± 1.14 g in the CT26 group, while the celecoxib group did not show an increase, with a tumor-free body weight of 20.63 ± 1.41 g (Figure 1E). These results were achieved without any effect on the tumor weight or food intake (Figures 1F, G). The CI group also exhibited a significant improvement in motility compared to the CT26 group, whereas the celecoxib group did not show a significant improvement (Figure 1H). Similarly, compared to the CT26 group, the CI group exhibited increases in TA and GAS muscle weights, while the celecoxib group showed no such increases. The spleen weight was reduced only in the celecoxib group (Figure 1I). Furthermore, treatment with CI or celecoxib reduced IL-6 level, one of the clinical indicators of cancer cachexia, in the serum compared to the CT26 group (Figure 1J).

### 3.2 CI improves glucose tolerance and muscle atrophy in a cancer cachexia mouse model

**FIGURE 2:**
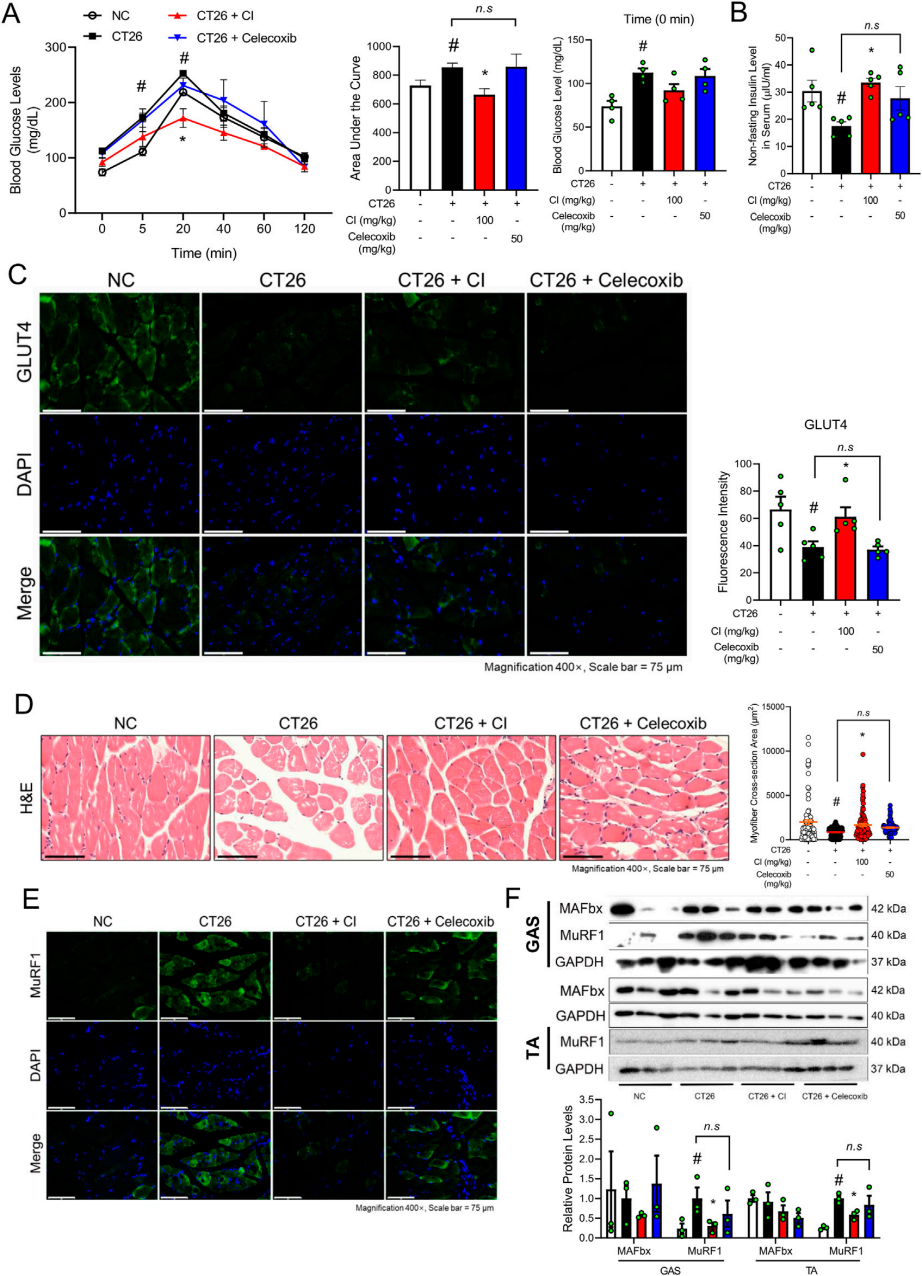
CI reduced the blood glucose levels and MuRF1 expression in CT26-induced cachectic BALB/c mice. **(A)** Oral glucose tolerance test was done, and the area under the curve was calculated (*n* = 4/group). The baseline serum glucose level was measured at 0 min and presented as a graph. **(B)** Non-fasting insulin level in serum was measured (*n* = 5/group). **(C)** GLUT4 (green) and nuclei (blue) were detected by immunofluorescence staining in GAS (×400 magnification, scale bar = 75 μm). Fluorescence intensity was quantified with the ImageJ software. **(D)** Paraffin-embedded GAS from the NC, CT26, and CI groups was stained with H&E (×400 magnification, scale bar = 75 μm). The myofiber cross-sectional area was measured with the ImageJ software. **(E)** MuRF1 (green) and nuclei (blue) were detected by immunofluorescence staining in GAS (×400 magnification, scale bar = 75 μm). **(F)** Protein levels of MAFbx and MuRF1 were measured by Western blot analysis in GAS and TA. Results were expressed relative to GAPDH. All data are expressed as the mean ± S.E.M. of three or more independent experiments. Statistical differences were calculated by two-way or one-way ANOVA and Tukey’s test. ^#^ *p* < 0.05 vs. NC mice; *p < 0.05 vs. CT26 mice. CI, *Chrysanthemum indicum*; NC, normal control; GAS, gastrocnemius; TA, tibialis anterior.

Given that insulin resistance has been identified as a significant contributing factor to cancer cachexia (Masi and Patel, 2021), we investigated whether CI affects glucose tolerance through the utilization of the OGTT. Mice injected with CT26 showed higher blood glucose levels compared to the NC group. Over the 120-min measurement period, the CI group exhibited blood glucose levels comparable to those of the NC group, whereas the celecoxib group demonstrated no discernible change compared to the CT26 group (Figure 2A). The CT26 group exhibited a decreased serum insulin level compared to the NC group, while the CI group showed an increase in insulin levels compared to the CT26 group. However, the celecoxib group showed no significant changes in the serum insulin level compared to the CT26 group (Figure 2B). Furthermore, the impact of CI on GLUT4, a pivotal factor in glucose tolerance, was also investigated. GLUT4 in the outer membrane of myofibers was highly expressed in the NC and CI groups, while the CT26 and celecoxib groups exhibited decreased GLUT4 expression (Figure 2C). Histological analysis revealed that the CI and celecoxib groups exhibited a markedly increased myofiber cross-section area in the GAS compared to the CT26 group (Figure 2D). The expression of key molecules associated with muscle atrophy, MuRF1 and MAFbx, was reduced in the CI group, whereas the celecoxib group did not show a reduction. In particular, the expression of MuRF1 was significantly reduced in the CI group (Figures 2E, F).

### 3.3 CI increases myofiber diameter and GLUT4 translocation in CT26 CM-treated C2C12 myofibers

**FIGURE 3:**
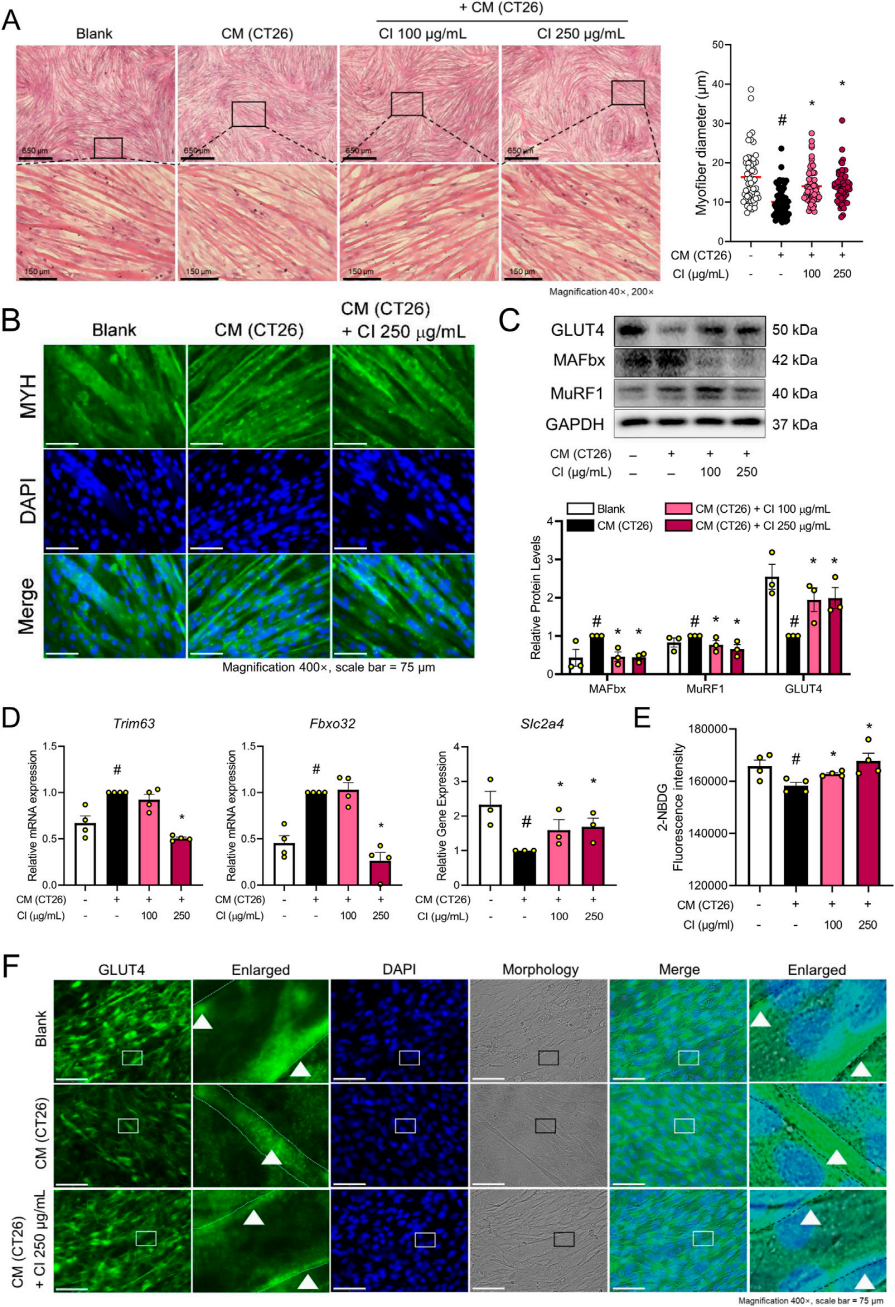
CI increased the myofiber diameter and glucose uptake through GLUT4 translocation in CM (CT26)-treated C2C12 cells **(A)** C2C12 myofibers were fixated with 10% formalin and stained with H&E (×40, ×200 magnification, scale bar = 650 μm, 150 μm, respectively). The myofiber diameters were measured with the ImageJ software. **(B)** MYH (green) and nuclei (blue) were detected by immunofluorescence staining (×200 magnification, scale bar = 150 μm). **(C)** Protein levels of GLUT4, MAFbx, and MuRF1 were measured by Western blot analysis. Results are expressed relative to GAPDH. **(D)** mRNA expressions of *Slc2a4, Fbxo32,* and *Trim63* were analyzed by qPCR. Results were expressed relative to *Gapdh*. **(E)** Glucose uptake assay was measured by 2-NBDG fluorescence. **(F)** GLUT4 (green) and nuclei (blue) were detected by immunofluorescence staining (×400 magnification, scale bar = 75 μm). All data are expressed as the mean ± S.E.M. of three or more independent experiments. Statistical differences were calculated using an unpaired *t*-test and a subsequent *post hoc* one-tailed Mann-Whitney *U* test. ^#^ *p* < 0.05 vs. Blank; *p < 0.05 vs. CM (CT26). CI, *Chrysanthemum indicum*; CM (CT26), CT26-derived conditioned medium.

The above results demonstrate that CI restores skeletal muscle mass and glucose tolerance in the *in vivo* experiments. To investigate the mechanisms by which CI regulates glucose tolerance and muscle atrophy, we used CT26 conditioned medium (CM)-treated C2C12 cells. The cytotoxicity of CI in C2C12 cells was assessed by treating the cells with CI at concentrations ranging from 1 to 1,000 μg/mL, followed by a WST-8 assay. CI treatment up to 250 μg/mL did not exhibit any cytotoxicity in the C2C12 cells (Supplementary Figure S1). Consequently, we selected concentrations of 100 and 250 μg/mL for further experiments. To visually represent the structure transformation and quantify the thickness of the myofibers, we conducted IF staining using the myosin heavy chain (MYH) antibody and H&E staining. CI treatment increased the myofiber diameters in a concentration-dependent manner compared to the CT26 CM treatment (Figure 3A). Similarly, IF staining for MYH showed that the CI treatment with 250 μg/mL restored the myofiber thickness (Figure 3B). The CI treatment also decreased the protein and gene expressions of MuRF1/*Trim63* and MAFbx/*Fbxo32*, while increasing the expressions of GLUT4/*Slc2a4* (Figures 3C, D). After confirming that CI was morphologically effective in improving muscle atrophy, we evaluated the glucose uptake efficiency with the 2-NBDG fluorescence assay. Following the administration of 2-NBDG, we observed decreased glucose uptake in the CT26 CM-treated C2C12 cells, while the CI treatment increased the glucose uptake in these cells (Figure 3E). As shown in Figure 3F, CI treatment increased the expression of GLUT4, which was reduced by the CM treatment. Notably, CI treatment enhanced the GLUT4 expression in the cell membranes of the myofibers.

### 3.4 Linarin increases GLUT4 translocation and inhibits MuRF1 in CT26 CM-treated C2C12 myofibers

**FIGURE 4:**
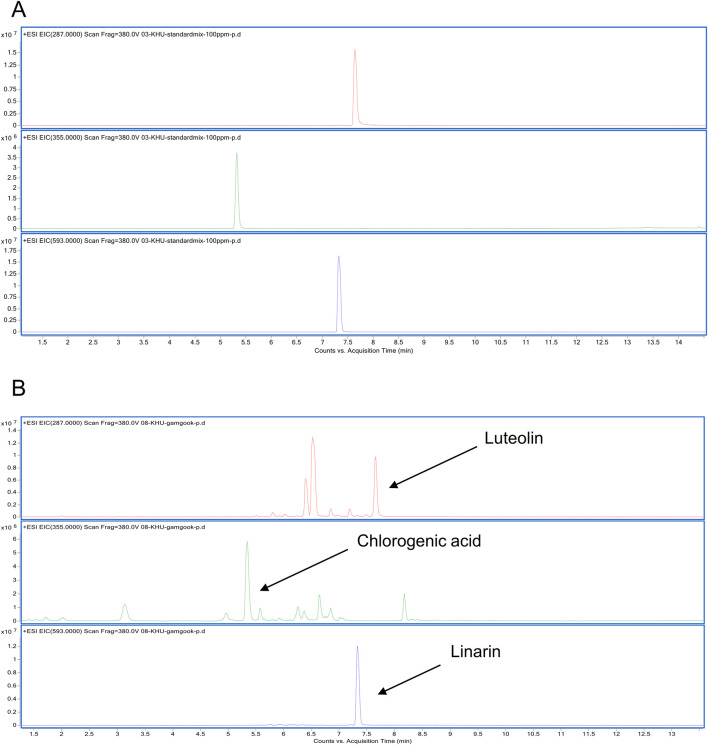
LC/MS analysis of CI **(A)** EIC of a cocktail of CI (Luteolin, Chlorogenic acid, Linarin) was analyzed with the ESI method. **(B)** EIC of the CI was obtained at m/z 287, m/z 355, m/z 593 respectively. CI, *Chrysanthemum indicum*; ESI, electrospray ionization; EIC, extracted ion chromatogram.

**FIGURE 5:**
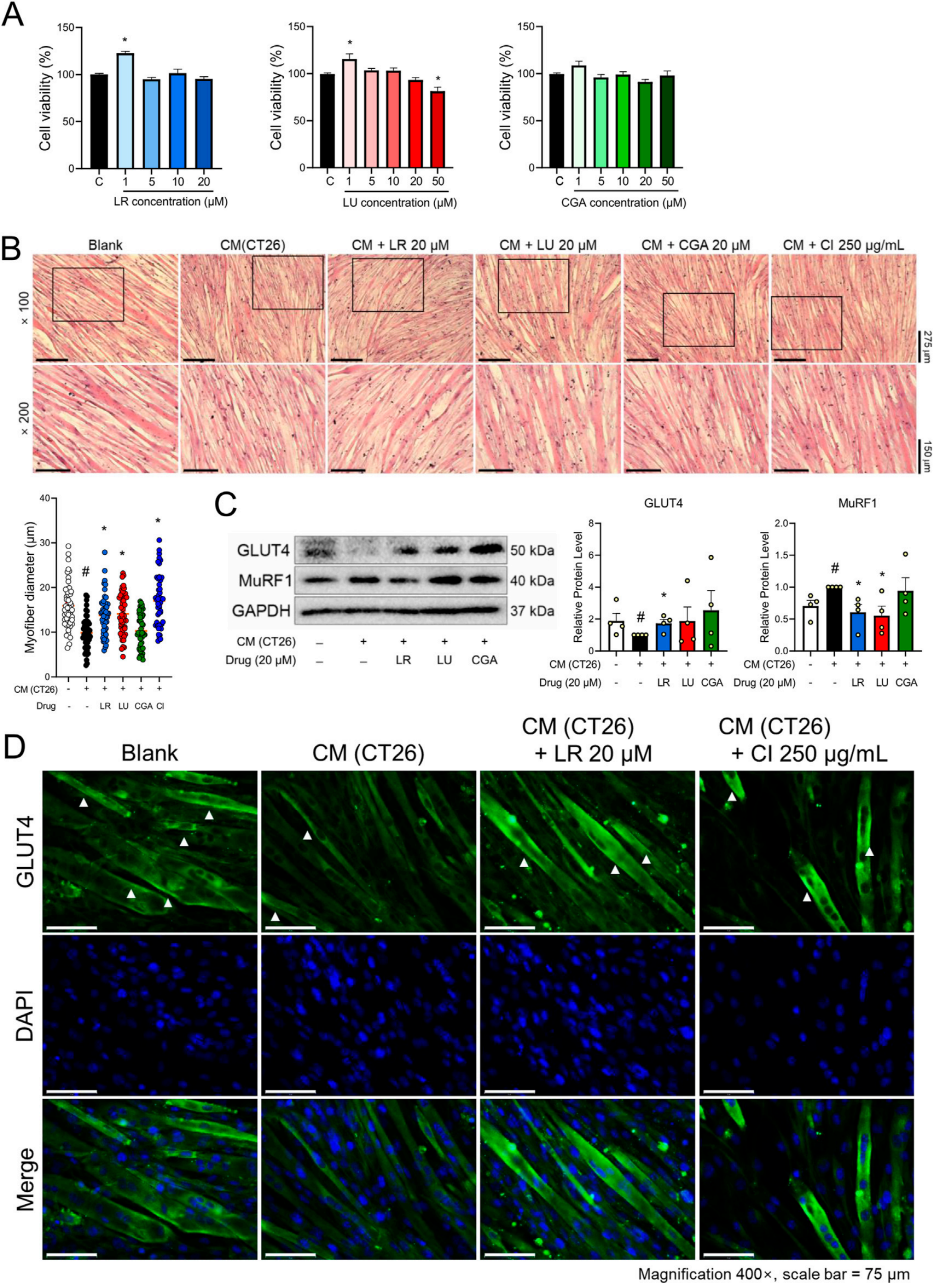
Components of CI increased myofiber thickness and GLUT4 expression in CM (CT26)-treated C2C12 cells. **(A)** Cytotoxicity of LR, LU, and CGA on C2C12 myoblasts were measured by the WST-8 assay. **(B)** C2C12 myofibers were fixated with 10% formalin and stained with H&E (×100, ×200 magnification, scale bar = 275 μm, 150 μm, respectively). The myofiber diameters was measured with the ImageJ software. **(C)** Protein levels of GLUT4 and MuRF1 were measured by Western blot analysis. Results are expressed relative to GAPDH. **(D)** GLUT4 (green), and nuclei (blue) were detected by immunofluorescence staining (×400 magnification, scale bar = 75 μm). All data are expressed as the mean ± S.E.M. of three or more independent experiments. Statistical differences were evaluated using an unpaired *t*-test and a subsequent *post hoc* one-tailed Mann-Whitney *U* test. ^#^ *p* < 0.05 vs. Blank; *p < 0.05 vs. CM (CT26). CI, *Chrysanthemum indicum*; LR, Linarin; LU, Luteolin; CGA, Chlorogenic acid; CM (CT26), CT26-derived conditioned medium.

An LC/MS analysis was done to provide a chemical profile of CI. The analysis revealed the presence of luteolin, chlorogenic acid, and linarin, previously reported as major constituents (Figure 4). Then, *in vitro* studies were done with luteolin, chlorogenic acid, and linarin to understand the active constituent of CI. Based on cell viability tests, we selected a concentration of 20 μM for luteolin, chlorogenic acid, and linarin (Figure 5A). As shown in Figure 5B, treatment with linarin and luteolin resulted in a similar increase in myofiber diameter to those observed with CI. Both linarin and luteolin treatments reduced the MuRF1 protein expression, and notably, linarin treatment significantly increased the GLUT4 expression (Figure 5C). Furthermore, linarin treatment resulted in an increase in GLUT4 expression in myofiber membranes to a level comparable to that observed in CI (Figure 5D). Consequently, we propose that CI, with linarin as an active compound, exerts a beneficial effect on muscle atrophy and glucose intolerance.

## 4 Discussion

The present study with the CT26-induced cancer cachexia model demonstrated that CI and its component linarin are effective in restoring glucose tolerance and alleviating skeletal muscle atrophy caused by cancer cachexia. Changes in weight alongside tumor growth are critical factors in cancer patients. While increased adiposity is plausibly associated with a higher risk of mortality, strong evidence also suggests that greater lean mass, particularly muscle, is linked to reduced risks (Bradshaw, 2024). Therefore, improving or alleviating cancer cachexia, particularly in cases of colon cancer, is crucial for enhancing the survival and overall prognosis of cancer patients.

Cachexia is a multifactorial syndrome that causes significant weight loss due to the loss of skeletal muscle and body fat in patients with advanced cancer. The prevalence of cancer cachexia is highest in pancreatic and stomach cancer, followed by colon cancer and lung cancer. The variation in the incidence of cancer cachexia is believed to arise from factors such as the type, location, and stage of the tumor (Bonetto et al., 2016; Ni and Zhang, 2020). For instance, cachexia occurs with higher frequency in cancers that are typically diagnosed at a more advanced stage, such as pancreatic and colon cancers. Additionally, the head and neck cancers, which may impair the ability to consume food, also exhibit a higher incidence of cachexia. Furthermore, differences in the expression of cytokines secreted by cancer cells may play a significant role in influencing the development and severity of cancer cachexia (Kasprzak, 2021). Among the various cancer cell lines, CT26 cells, derived from colon carcinoma, are the most commonly used in cancer-induced cachexia *in vivo* experiments due to their ability to reproducibly cause muscle and adipose tissue loss compared with other carcinoma mouse models (Bonetto et al., 2016; Neyroud, Nosacka et al., 2019). Cancer cachexia is characterized by a number of metabolic changes, including insulin resistance, muscle atrophy and inflammatory responses. Potential treatments for cancer cachexia include appetite stimulants, anabolic agents, cytokine inhibitors, insulin, NSAIDs, and others (Lundholm et al., 2004a; Lundholm et al., 2004b; Roeland et al., 2020; Roeland et al., 2023). Celecoxib, a cyclooxygenase-2 (COX-2) selective inhibitor included in NSAIDs, has been reported to have an anti-cachectic effect in CT26 tumor-bearing mice (Xu et al., 2015). Celecoxib has demonstrated enhanced anticancer effects when used in combination with 5-FU, cisplatin, and folinic acid (Choi et al., 2021; Köhne et al., 2008; Liu et al., 2017; McCrohan et al., 1987; Sung et al., 2017). Studies on celecoxib in patients with cancer cachexia have demonstrated that it is safe to use at the tested doses (200–400 mg/day). However, conflicting results regarding its efficacy have been reported, which may reflect heterogeneity among the studies (Bowers et al., 2023). In our study, celecoxib effectively reduced serum IL-6 levels and NF-κB but was less effective in suppressing cancer cachexia (Figure 1; Supplementary Figure S2). However, there was no increase in the tumor-free body weight and TA weight compared to CT26-induced cachexia mice. However, the CI treatment was found to improve cancer cachexia parameters, including tumor-free body weight, inflammation cytokine, muscle activity, and skeletal muscle weights including GAS and TA (Figure 1A). These results indicate that inflammation suppression alone is insufficient for effectively treating cancer cachexia. Additionally, chemotherapy agents such as 5-FU and folinic acid can induce cancer cachexia, particularly at high doses (Pin et al., 2019; VanderVeen et al., 2024; Wang J. et al., 2022). 5-FU and folinic acid are widely used treatments for patients with colorectal cancer. If administered in conjunction with CI, it is anticipated that this combination could positively impact the survival rates of colorectal cancer patients by addressing muscle atrophy alongside their cancer-suppressive effects.

The loss of skeletal muscle mass represents a pivotal element in the pathophysiology of cancer cachexia (Evans, 2010; Evans et al., 2008). This loss is typically attributed to a reduction in protein synthesis, an increase in protein degradation, or a relative imbalance between synthesis and degradation (Fearon et al., 2012). Protein synthesis is regulated by activating the IGF-1/PI3K/Akt/mTOR signaling pathway (Yin et al., 2021). This pathway operates through two primary substrates in response to relevant stimuli: one is eukaryotic initiation factor 4E-binding protein 1 (4E-BP1), whose phosphorylation prevents the inactivation of eukaryotic initiation factor 4E (eIF4E); the other is 70 kDa ribosomal protein S6 kinase 1 (S6K1), which, when phosphorylated, regulates ribosomal protein S6 and eukaryotic elongation factor 2, thereby promoting downstream mRNA translation. However, CI treated mice did not restore the reduced expression of 4E-BP1 observed in cancer-injected mice (Supplementary Figure S3). The UPS, which is known to be heavily involved in the muscle-wasting process, serves as the primary regulatory mechanism of protein degradation in skeletal muscle (Rom and Reznick, 2016). Representative UPS proteins, such as MAFbx/*Fbxo32* and MuRF1/*Trim63*, have been shown to be highly active in cancer cachexia (Yang et al., 2020). In our study, CI significantly reduced the expression of the UPS components, particularly MuRF1, in both the *in vivo* and *in vitro* cachexia models. MuRF1 and MAFbx are regulated by various signaling pathways such as PI3K, Akt, NF-κB, and IL-6, signaling pathways (Cai et al., 2004; Li et al., 2009; Li et al., 2005). Studies have demonstrated that NF-κB can regulate the expression of MuRF1; however, it does not exert the same regulatory effect on MAFbx (Glass, 2005). Interestingly, our results indicate that an increase in NF-κB was associated with MuRF1 in GAS (Supplementary Figure S2). While MuRF1 is most commonly associated with its role in sarcomeric protein degradation, recent findings suggest that it may also have broader signaling effects on insulin homeostasis and glucose metabolism (Bodine and Baehr, 2014; Labeit et al., 2021; Scalabrin et al., 2020). Skeletal muscle is one of the key organs involved in insulin metabolism, and protein synthesis and degradation in muscle are regulated by the Insulin-like growth factor 1 (IGF-1)/phosphatidylinositol 3-kinase (PI3K)/Akt pathway (Lee et al., 2022). Abnormal glucose metabolism is associated with cancer cachexia and leads to altered metabolic states, such as elevated plasma glucose and insulin resistance (Asp et al., 2010; Masi and Patel, 2021). Insulin stimulation induces glucose consumption primarily in skeletal muscle, thereby affecting systemic metabolism (Lee et al., 2022). Numerous studies on cancer cachexia have demonstrated a strong association between insulin resistance and muscle atrophy (Honors and Kinzig, 2012; Jafri et al., 2015). Furthermore, muscle atrophy linked to insulin resistance is also observed in other conditions related to sarcopenia, such as diabetes and obesity-induced muscle atrophy. In contrast to the high-fat diet (HFD) model, which typically requires 10 weeks to 5 months for significant muscle atrophy and insulin resistance to develop, cancer cachexia induces insulin resistance within a much shorter period, often as early as 2–4 weeks (Bonetto et al., 2016; Chae et al., 2023; Guo et al., 2021; Ning et al., 2023). This difference is likely due to variations in the underlying mechanisms of muscle atrophy as well as differences in the experimental models. Cancer studies generally conclude within 2–5 weeks following the injection of cancer cell, while obesity models using HFD necessitate at least 8 weeks of HFD administration. Nevertheless, it is evident that insulin resistance and muscle atrophy are closely interconnected.

In this context, CI demonstrated an improvement in glucose tolerance, non-fasting insulin serum levels, and an increase in GLUT4 expression, accompanied by a reduction in skeletal muscle atrophy, which may explain these findings. In particular, CI was observed to increase GLUT4 translocation to the plasma membrane in C2C12 myofibers (Figure 3). GLUT4 is a transporter that facilitates glucose uptake in muscle fibers upon insulin stimulation. The enhanced translocation of GLUT4 to the plasma membrane in skeletal muscles has been shown to improve insulin sensitivity (Mueckler, 2001). However, the limitation of our study is that insulin sensitivity could not be directly assessed due to the absence of an insulin tolerance test. Nevertheless, the lower circulating insulin levels and higher fasting glucose levels observed in the CT26 group may suggest impaired insulin responsiveness, implying that CI could potentially ameliorate this condition. Insulin mediates GLUT4 translocation through two distinct pathways: the insulin receptor substrate (IRS)/PI3K/Akt pathway and the APS-dependent insulin signaling pathway (Gonzalez and McGraw, 2006; Sayem et al., 2018). In our study, CI was found to enhance the APS-dependent pathway by upregulating key regulators of GLUT4 translocation, including phosphorylated AKT (pAKT), *Sorbs1, Crk,* and *Trip10* (Supplementary Figure S4). While the IRS-PI3K-Akt pathway is well-established for its role in promoting glucose uptake in skeletal muscle, its activation does not fully replicate the efficiency of insulin signaling, particularly with respect to GLUT4 translocation (Ng et al., 2008; Sayem et al., 2018). Recent studies have increasingly highlighted the significance of the APS-dependent pathway (Moodie et al., 1999).

APS, a novel insulin receptor substrate protein, is predominantly expressed in insulin-responsive tissues and is essential for insulin-stimulated glucose uptake and GLUT4 translocation via the CAP/*Sorbs1*-Cbl-*Crk*-TC10 complex. Alteration of APS expression is implicated in the pathogenesis of insulin resistance, particularly in skeletal muscle (Rea et al., 2005). Upon insulin receptor activation, APS is phosphorylated, initiating downstream signaling that activates the small GTPase TC10 within lipid rafts. This activation regulates cellular processes such as actin cytoskeleton remodeling, recruitment of effectors like CIP4/*Trip10*, and assembly of the exocyst complex, which are essential mechanisms for proper trafficking and recycling of GLUT4 vesicles (Chang et al., 2004; Liu et al., 2002; Sayem et al., 2018). While our data indicate that CI modulates the APS-dependent pathway, further research is required to fully elucidate the upstream molecular mechanisms by which CI regulates GLUT4 translocation. These findings indicate that CI may be a promising therapeutic approach for improving both skeletal muscle atrophy and glucose tolerance in the context of cancer cachexia.

Additionally, CI was recovered glucose uptake as well as the oxygen consumption rate *in vitro* model (Figure 3E; Supplementary Figure S5). The transformation of glucose utilization from oxidative phosphorylation to glycolysis is now regarded as a major feature of cancer. This kind of change in energy metabolism is regulated by genetic changes and tumor microenvironment pressure, causing an increase in the cell proliferation rate and conferring resistance on cell (Abbaszadeh et al., 2020; Liu et al., 2022). Although limited studies exist on the efficacy of CI in colon cancer, research on other cancer types has demonstrated that CI inhibits proliferation and metastasis (Kim et al., 2013; Wang et al., 2010; Yuan et al., 2009). Additionally, CI slightly reduced tumor size and weight, although not statistically significant. When these findings are considered collectively, it is possible that CI modulates glucose homeostasis, thus affecting colon cancer progression.

Furthermore, our findings indicate that the pharmacological effect of CI in improving cancer cachexia is primarily attributed to linarin among its many components (Figure 5). Recent studies have demonstrated that extracts or monomeric compounds derived from CI exhibit a range of pharmacological activities, including anti-inflammatory, anti-oxidative, antipathogenic microbial, anticancer, immune regulatory, and hepatoprotective effects (Shao et al., 2020; Wu et al., 2013). LC/MS analysis revealed that linarin, luteolin, and chlorogenic acid are the major components present in CI in significant quantities (Figure 4). Previous studies have demonstrated that linarin regulates glucose homeostasis in diabetes (Chenafa et al., 2022; Wang et al., 2023; Wang Z. et al., 2022). Furthermore, chlorogenic acid has been shown to reduce the risk of developing diabetes and prevent blood glucose spikes (Faraji, 2018; Yanet al., 2022). Luteolin has been observed to improve muscle atrophy in models of cachexia and sarcopenia induced by LLC and obesity, respectively (Chen et al., 2018; Kim et al., 2023). In the present study, both linarin and luteolin significantly reduced the expression of MuRF1 and increased muscle thickness. In particular, linarin significantly increased GLUT4 translocation to the plasma membrane and the protein expression of GLUT4, a result comparable to that observed with CI (Figure 5). These findings suggest that linarin may be an active ingredient in CI, contributing to its effectiveness in treating cancer cachexia. Comprehensively, CI represents a promising novel alternative treatment for cancer cachexia, addressing major symptoms such as glucose intolerance and muscle atrophy.

In conclusion, CI improves cancer-induced muscle atrophy and glucose intolerance through GLUT4 translocation and MuRF1 signaling in the CT26-induced cancer cachexia animal and cell models. These findings advance understanding of the pharmacological role of CI in the treatment of cancer cachexia.

## Data Availability

The original contributions presented in the study are included in the article/Supplementary Material, further inquiries can be directed to the corresponding authors.
