# Supplementary material for: Chrysanthemum indicum L. ameliorates muscle atrophy by improving glucose tolerance in CT26-induced cancer cachexia
Source: Front Pharmacol. 2024 Nov 18;15:1455805. doi: 10.3389/fphar.2024.1455805 (PMC11609647; doi:10.3389/fphar.2024.1455805)
Supplement: Supplementary file 1 [file DataSheet1.docx]

***Supplements information***

***Chrysanthemum Indicum* L*.* ameliorates muscle atrophy by improving glucose tolerance in CT26-induced cancer cachexia**

Song et al.

*Table S1. Antibodies information*

*Table S2. Primer information*

*Figure S1. CI on WST-8 assay in C2C12 cells*

*Figure S2. CI decreased NF-κB in GAS of CT26-induced cachectic BALB/c mice*

*Figure S3. CI did not change 4E-BP1 in GAS of CT26-induced cachectic BALB/c mice*

*Figure S4. CI induced AKT-independent glucose uptake pathway in CM (CT26)-treated C2C12 cells*

*Figure S5. CI increased extracellular oxygen consumption in CM (CT26)-treated C2C12 cells*

**Table S1. Antibodies information**

| Category | Name of antibody | Company | Catalog No. | Molecular weight (kDa) | Experiments |
| --- | --- | --- | --- | --- | --- |
| Primary antibody | GLUT4 | Cell signaling technology | 2213s | 50 | WB, IF |
|  | pAKT (Ser473) |  | 4060 | 60 | WB |
|  | AKT |  | 4691s | 60 | WB |
|  | 4E-BP1 |  | 9452s | 15 to 20 | IF |
|  | MuRF1 | GeneTex | GTX33334 | 40 | WB |
|  | MAFbx | Santa Cruz Biotechnology | sc-166806 | 42 | WB |
|  | MYH |  | sc-376157 | 200 | IF |
|  | NF-κB |  | Sc-372 | 65 | IF |
|  | GAPDH |  | sc-32233 | 37 | WB |
| Secondary antibody | Goat anti Rabbit IgG (HRP) | GeneTex | GTX213110 |  | WB |
|  | Goat anti Mouse IgG (HRP) |  | GTX213111 |  | WB |
|  | Goat anti-Rabbit IgG (H+L) Alexa Fluor™ 488 | Invitrogen | A-11008 |  | IF |
|  | Goat anti-Mouse IgG (H+L) Alexa Fluor™ 488 |  | A-28175 |  | IF |

GLUT4, glucose transporter type 4; 4E-BP1, Eukaryotic translation initiation factor 4E-binding protein 1; MuRF1, muscle RING-finger protein-1; MAFbx, muscle atrophy F-box; MYH, myosin heavy chain; NF-κB, Nuclear factor kappa-light-chain-enhancer of activated B cells; GAPDH, glyceraldehyde 3-phosphate.

**Table S2. Primer information**

| **Gene name** | **Forwards (5’ → 3’)** | **Reverses (5’ → 3’)** |
| --- | --- | --- |
| *Fbxo32* | CAGCTTCGTGAGCGACCTC | GGCAGTCGAGAAGTCCAGTC |
| *Trim63* | GTGTGAGGTGCCTACTTGCTC | GCTCAGTCTTCTGTCCTTGGA |
| *Slc2a4* | GTGACTGGAACACTGGTCCTA | CCAGCCACGTTGCATTGTAG |
| *Sorbs1* | GATGAGTCGGGATATAAGCCCA | GAGCAGTCTCCAGGAGTATAGTC |
| *Crk* | GGAGGTCGGTGAGCTGGTA | CGTTTGCCATTACACTCCCCT |
| *Trip10* | CCACACTGTCTGATGGACCC | ACCGACTTACTGATCTCGCTC |
| *Gapdh* | AGGTCGGTGTGAACGGATTTG | TGTAGACCATGTAGTTGAGGTCA |

*Fbxo32*, muscle atrophy F-box; *Trim63*, muscle RING-finger protein-1; *Slc2a4*, solute carrier family 2 (facilitated glucose transporter), member 4; *Sorbs1,* SH3 domain-containing protein 1*; Crk,* CRK proto-oncogene, adaptor protein; *Trip10,* thyroid hormone receptor interactor 10*; Gapdh*, glyceraldehyde 3-phosphate.

**Figure S1. CI on WST-8 assay in C2C12 cells**

C2C12 myoblasts were incubated with CI at the indicated concentrations (1-1000 μg/mL), and cell viability after 24 h was assessed by the WST-8 assay. All data are expressed as the mean ± S.E.M. of three or more independent experiments. Statistical differences were evaluated using an unpaired *t*-test and a subsequent *post hoc* one-tailed Mann-Whitney *U* test. ^*^*p* < 0.05 vs. Control. CI, *Chrysanthemum indicum.*


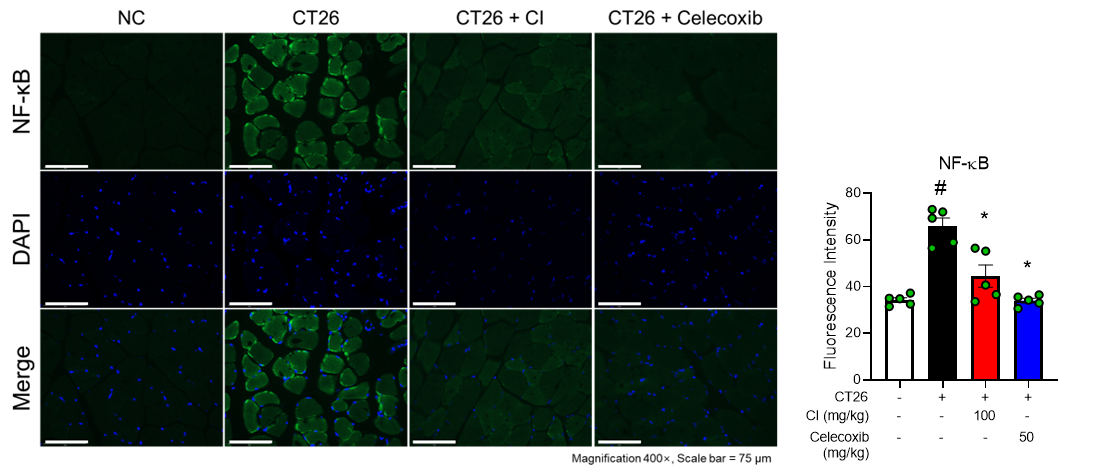


**Figure S2. CI decreased NF-κB in GAS of CT26-induced cachectic BALB/c mice**

NF-κB (green) and nuclei (blue) were detected by immunofluorescence staining in GAS (400× magniﬁcation, scale bar = 75 μm). Fluorescence intensity was quantified with the ImageJ software. All data are expressed as the mean ± S.E.M. of five independent experiments. Statistical differences were calculated by one-way ANOVA and Tukey’s test. ^#^*p* < 0.05 vs. NC mice; ^*^*p* < 0.05 vs. CT26 mice. CI, *Chrysanthemum indicum*; NC, normal control; GAS, gastrocnemius.


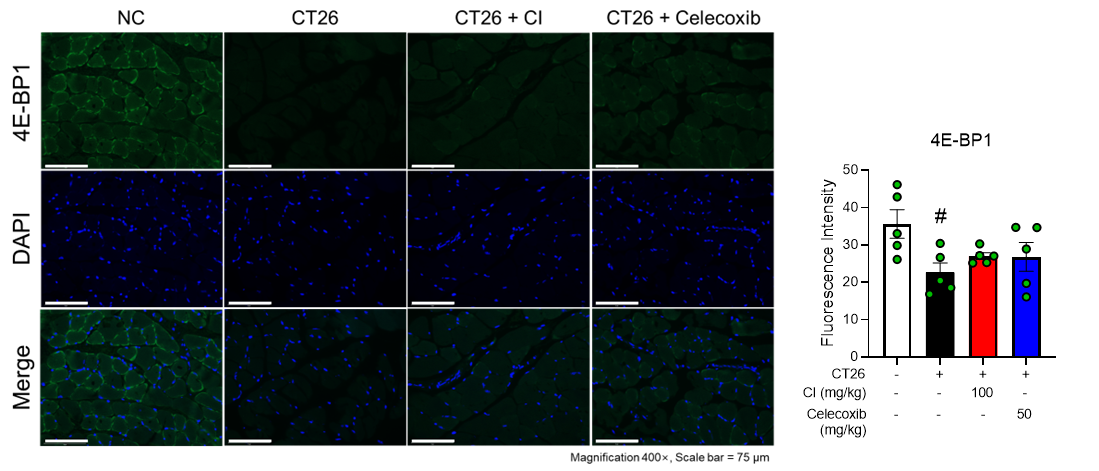


**Figure S3. CI did not change 4E-BP1 in GAS of CT26-induced cachectic BALB/c mice**

4E-BP1 (green) and nuclei (blue) were detected by immunofluorescence staining in GAS (400× magniﬁcation, scale bar = 75 μm). Fluorescence intensity was quantified with the ImageJ software. All data are expressed as the mean ± S.E.M. of five independent experiments. Statistical differences were calculated by one-way ANOVA and Tukey’s test. ^#^*p* < 0.05 vs. NC mice; ^*^*p* < 0.05 vs. CT26 mice. CI, *Chrysanthemum indicum*; NC, normal control; GAS, gastrocnemius.


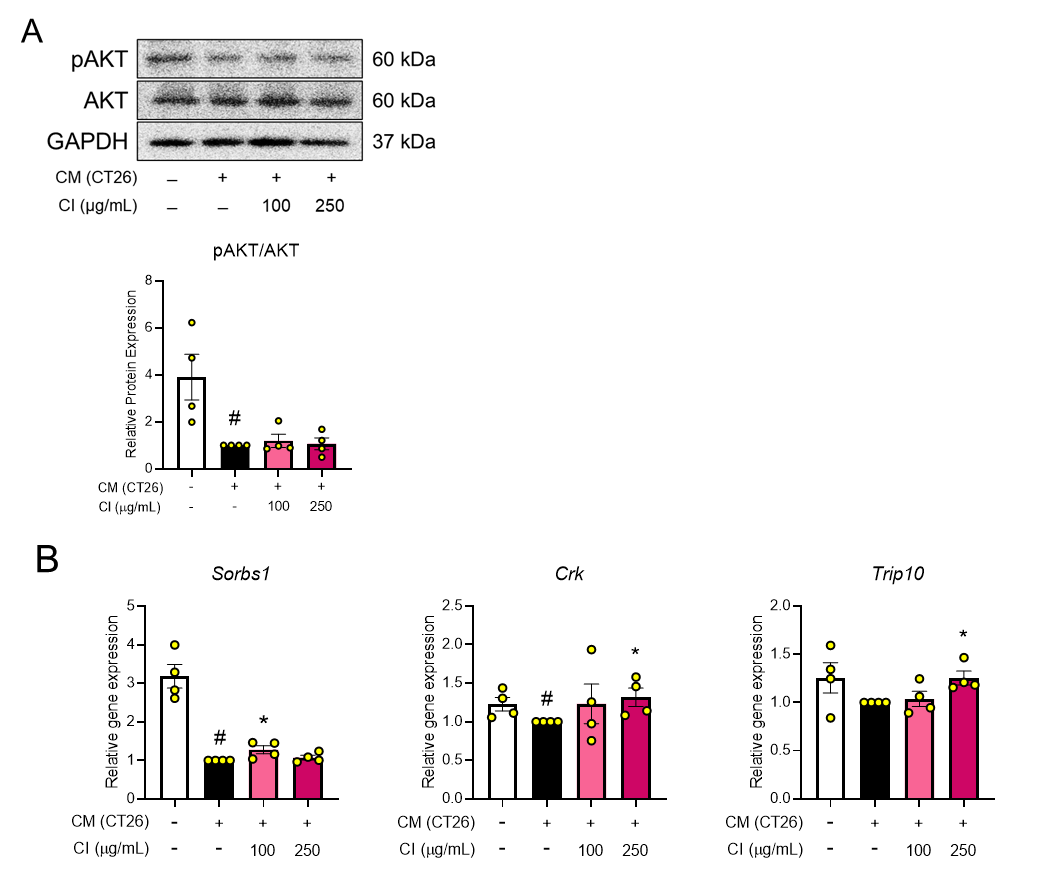


**Figure S4. CI induced AKT-independent glucose uptake pathway in CM (CT26)-treated C2C12 cells**

(A) Protein level of pAKT were measured by Western blot analysis. Results are expressed relative to AKT. (B) mRNA expressions of *Sorbs1, Crk*, and *Trip10* were analyzed by qPCR. Results were expressed relative to *Gapdh.* All data are expressed as the mean ± S.E.M. of four independent experiments. Statistical differences were calculated using an unpaired *t*-test and a subsequent *post hoc* one-tailed Mann-Whitney *U* test. ^#^*p* < 0.05 vs. Blank; ^*^*p* < 0.05 vs. CM (CT26). CI, *Chrysanthemum indicum*; CM (CT26), CT26-derived conditioned medium.


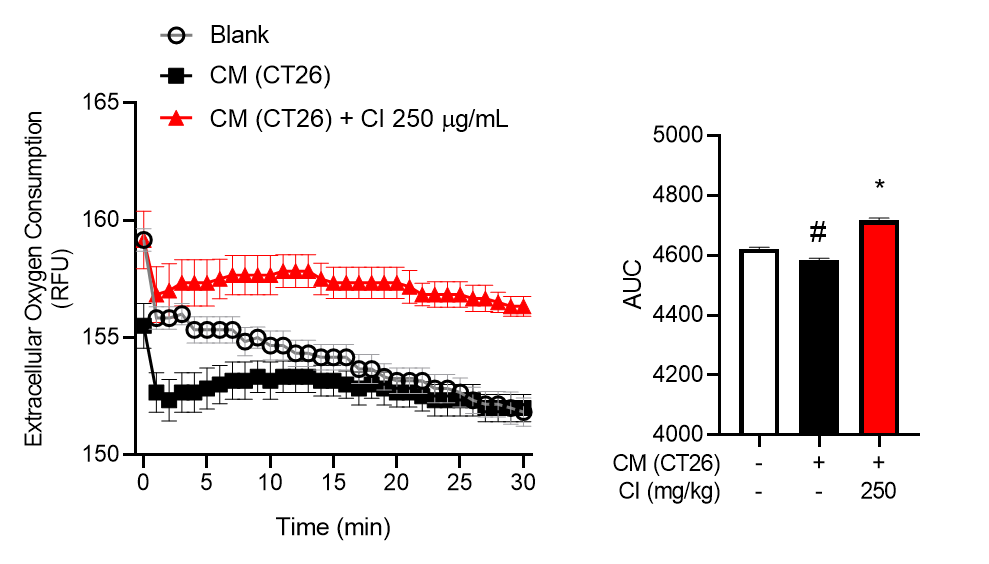


**Figure S5. CI increased extracellular oxygen consumption in CM (CT26)-treated C2C12 cells**

Extracellular oxygen consumption was analyzed, and the area AUC was calculated. All data are expressed as the mean ± S.E.M. of five independent experiments. Statistical differences were calculated by two-way or one-way ANOVA and Dunnett's test. ^#^*p* < 0.05 vs. Blank; ^*^*p* < 0.05 vs. CM (CT26). CI, *Chrysanthemum indicum*; CM (CT26), CT26-derived conditioned medium.
